# Outcomes of reverse shoulder arthroplasty as a day case procedure: a population-based cohort study using the National Joint Registry and Hospital Episode Statistics

**DOI:** 10.1308/rcsann.2025.0035

**Published:** 2025-06-17

**Authors:** O O’Malley, A Davies, A Rangan, S Sabharwal, P Reilly

**Affiliations:** ^1^ Cutrale Perioperative & Ageing Group, UK; ^2^ University of York, UK; ^3^ Imperial College Healthcare NHS Trust, UK

**Keywords:** Reverse shoulder arthroplasty, Day case, Day case arthroplasty

## Abstract

**Introduction:**

Reverse shoulder arthroplasty (RSA) is the most common shoulder replacement in the United Kingdom and has traditionally been an inpatient procedure. Advances in anaesthetic and surgical techniques have made day case RSA increasingly popular, yet published data on its outcomes are limited. This study assesses the outcomes of day case RSA using the National Joint Registry (NJR).

**Methods:**

NJR data (April 2012–March 2022) were linked with Hospital Episode Statistics. All patients undergoing RSA for any indication were included. Primary outcomes were revision surgery rates; secondary outcomes included non-revision reoperation, medical complications requiring readmission and patient-reported outcomes for day case vs non-day case patients.

**Results:**

Among 320 day case and 25,748 non-day case RSA patients, day case patients were younger, predominantly male and had lower comorbidity scores. Day case revision rates were 1.45% at 1 year, 1.93% at 3 and 5 years, and 3.96% at 7 years compared with 1.76%, 2.84%, 3.53% and 4.35% for non-day cases. Readmissions for medical complications occurred in 1.56% of day case patients vs 6.34% of non-day cases. Mean Oxford Shoulder Score improvements were 19.83 (±11.32) and 19.16 (±11.80) for day and non-day case groups, respectively.

**Conclusion:**

Day case RSA demonstrates low revision, reoperation and complication rates, with similar patient satisfaction to non-day cases. These findings highlight the safety of day case RSA with careful patient selection and its potential to improve healthcare efficiency from a policy perspective.

## Introduction

Reverse shoulder arthroplasty (RSA) is now the most common shoulder arthroplasty used in both the United Kingdom (UK) and the United States.^1,2^ It has traditionally been an inpatient procedure with the average length of stay being 1.31 to 2.8 days.^3^ With increasing pressure on healthcare systems as well as increasing costs associated with a prolonged length of stay, day case arthroplasty surgery has seen an increasing trend.^4,5^

There is limited literature on day case surgery in RSA. One UK study reviewed 21 patients who underwent day case RSA surgery and reported high patient satisfaction and no readmissions; however, the sample size was small with a limited follow-up of 12 months.^3^ An earlier study by Fournier *et al* reviewed 61 day case shoulder arthroplasty patients, only 12 of whom were RSA cases.^6^ The authors concluded that day case surgery had a low rate of perioperative complications with no readmissions; however, their sample size was also small and there were no long-term outcomes.

This study aims to utilise the National Joint Registry (NJR) to report on the outcomes of day case RSA surgery in England, Wales, Northern Ireland, the Isle of Man and the State of Guernsey, based on revision rate, non-revision secondary surgery, medical complications requiring readmission and patient outcomes.

## Methods

### Data source

Data were requested from the NJR, which has been routinely collecting shoulder arthroplasty data since 2012 in England, Wales, Northern Ireland, the Isle of Man and the State of Guernsey. The NJR data set includes patient demographics, operation details and patient outcome in terms of revision, unrevised or death. Health Episode Statistics (HES) data contain records of every patient admission to a National Health Service (NHS) hospital in England and publicly funded admissions in independent hospitals, and include data such as medical diagnosis in the form of International Classification of Diseases (ICD-10) codes and operation data in the form of OPCS 4.9 codes. These two data sets can be linked to the Office for National Statistics mortality register, which is regularly updated and linked to HES data.

### Inclusion

All patients receiving an RSA for both elective indications and trauma included in the NJR between 1 April 2012 and 31 March 2022 were included in the study. The NJR database was linked to the HES database using the NJR index number. Length of stay was calculated from the primary procedure date in the NJR to the discharge date provided by HES. Day cases represented those with a length of stay of 0 days.

### Outcomes

The primary outcome is revision, defined as any operation in which one or more components are added to, removed from or modified in a joint replacement, or if a debridement and implant retention with or without modular exchange is performed.^2^ The first revision is recorded in the NJR data file. Secondary outcomes are reoperations, medical complications within 30 days of the procedure requiring readmission and patient-reported outcome in the form of Oxford Shoulder Score (OSS). To identify reoperation procedures, OPCS 4.9 codes were used (Appendix 1 – available online). Reoperations on the same shoulder not as part of a revision procedure were included. Reoperations included stabilisation, relocation, manipulation under anaesthesia (MUA) with or without capsular release, synovectomy and fracture fixation. Postoperative medical complications were identified from HES data, using ICD-10 codes and occurring within 30 days of the operative procedure (Appendix 2 – available online).^7^ The Charlson Comorbidity Index (CCI) score was calculated using ICD-10 codes from the HES database. Comorbidities were identified from episodes prior to or at the same time as the index arthroplasty (Appendix 3 – available online).^8–10^

Collection of patient-reported outcome measures (PROMs) in the form of the OSS is coordinated by the NJR. The OSS is a patient-focused, shoulder-specific questionnaire. It consists of 12 domains that are scored from 0 to 4 with a maximum score of 48.^11,12^ The OSS has been extensively tested and validated and is deemed reliable for assessing patients’ shoulder symptoms.^13^ The OSS is recorded preoperatively, and at 6 months, 3 and 5 years. As defined by Dawson *et al*, within the questionnaire if up to two items are missing, the average of the remaining items can be substituted for the missing values. If more than two items are missing, the results will be disregarded.^12^ Because of recall bias in concordance with NJR, the preoperative score must be within 90 days prior to the primary surgery until the day of the primary operation. Six-month data collection must take place within 5 to 8 months postoperatively. The 3- and 5-year data collections must be within 1 month prior to 3 and 5 years, respectively, to 6 months after.^2^ A month was taken to be an average month (30.42 days) to calculate cut-offs when validating the questionnaires. For the OSS, the minimally clinically important difference (MCID) for RSA at 6 months postoperatively has been calculated as 4.7 and this value is used in this study.^14^

### Ethical review

This study used pseudo-anonymised, routinely collected data from an established clinical registry, and patient consent is obtained by the NJR. According to Health Research Authority guidance, ethical approval was not required.

### Statistical analysis

The demographics of the groups are presented with descriptive statistics. A chi-squared test was used to determine statistical difference between categorical variables and a paired *t*-test or Mann–Whitney *U* test was used for continuous variables. Demographics were compared at the population level, at individual centres that completed more than ten day cases and in centres that do and do not complete day case surgery. The Kaplan–Meier method was used to analyse time to event data for implant survival at 1, 3, 5, 7 and 10 years, and 95% confidence intervals were calculated. Descriptive statistics for reoperation, medical complications and OSS score are presented. Statistical analysis was performed using StataSE v16 (StataCorp, USA). Strengthening the reporting of observational studies in epidemiology (STROBE) guidelines were adhered to in this study.^15^

## Results

**Figure 1:**
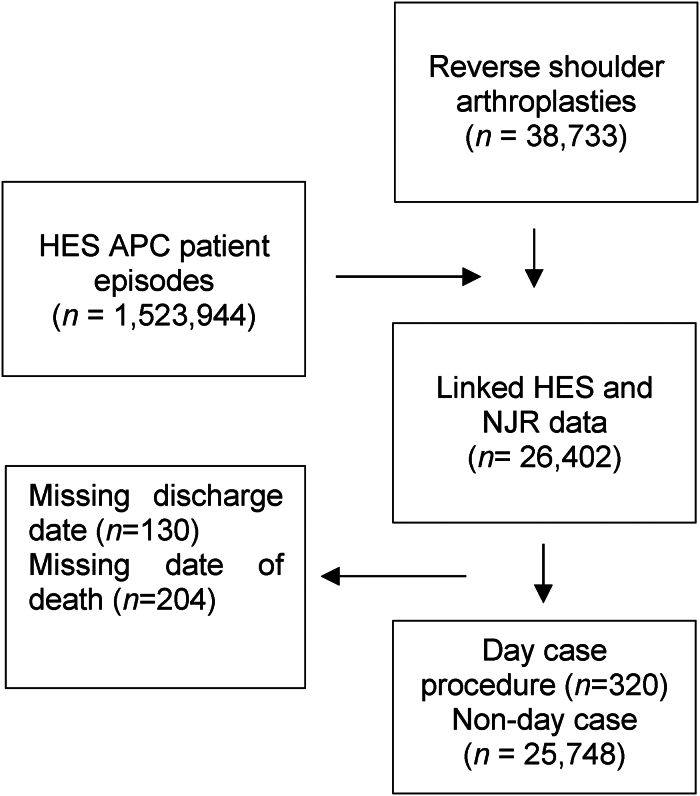
Flow chart of cohort

There were 320 patients who had a day case RSA between April 2012 to March 2022 and 25,748 non-day case patients (Figure 1). Patient demographics are presented in Table 1. The day case patients were younger, more likely to be male, although a majority of females in both groups persists, have a lower American Society of Anesthesiologists (ASA) and a lower comorbidity score than those in the non-day case group. There was no significant difference in deprivation index between the two groups.

**Table 1:** Patient demographics in day case and non-day case patients

Demographic	Day case	Non-day case	*p-*value
Age (sd)	73.23 (7.34)	75.35 (7.84)	<0.001*
Gender (%)			
Male	122 (38.1)	7,057 (27.54)	**<0.001**
Female	198 (61.9)	18,691 (72.6)	
Operative year (%)			
2012	1 (0.3)	686 (2.7)	**<0.001**
2013	10 (3.1)	1315 (5.1)	
2014	17 (5.3)	1,828 (7.1)	
2015	11 (3.4)	2,196 (8.5)	
2016	25 (7.8)	2,755 (10.7)	
2017	29 (9.1)	3,302 (12.8)	
2018	43 (13.4)	3,673 (14.3)	
2019	44 (13.8)	4,180 (16.2)	
2020	37 (11.6)	2,101 (8.2)	
2021	88 (27.5)	2,941(11.4)	
2022	15 (4.7)	771 (3.0)	
ASA (%)			
1	19 (5.9)	984 (3.8)	**0.004**
2	215 (67.2)	15,366 (59.7)	
3	85 (26.6)	9,095 (35.3)	
4 + 5	1 (0.3)	303 (1.2)	
Mean CCI (sd)	4.56 (2.01)	4.13 (1.84)	<0.001**
Deprivation group (%)			
I Most deprived	50 (15.6)	3,631 (14.1)	0.462
II	60 (18.8)	4,420 (17.2)	
III	65 (20.3)	5,892 (22.9)	
IV	114 (35.6)	8,792 (34.1)	
V Least deprived	30 (9.4)	2,697 (10.5)	
No deprivation data	1 (0.3)	316 (1.2)	

ASA = American Society of Anesthesiologists; CCI = Charlson Comorbidity Index; SD = Standard Deviation

*t-test

**Mann–Whitney *U* test.

In total, 362 centres in the database undertook RSA, 110 of which performed day case RSA (30.39%). Centres that offered day case surgery had a higher proportion of male patients (*p* = 0.001), a lower comorbidity score (*p* = 0.028) and were more likely to be in a less-deprived area (*p* < 0.001). There were no differences in patient age or ASA score between the centre types (Table 2). In these 110 centres, the case load of day case surgery ranged from 0.31% to 7.86%, with the largest centre completing 25 day case RSAs.

**Table 2:** Patient demographics in day case and non-day case centres

Demographic	Day case centre (*n* = 110)	No day cases performed (*n* = 252)	*p*-value
Age (sd)	75.28 (7.67)	75.37 (7.99)	0.320*
Gender (%)			
Male	3,499 (28.51)	3,680 (26.67)	**0** **.** **001**
Female	8,773 (71.49)	10,116 (73.33)	
ASA (%)			
1	469	534	0.529
2	7,281	8,300	
3	4,380	4,800	
4 + 5	142	162	
Mean CCI (sd)	4.54 (2.05)	4.58 (2.07)	**0**.028**
Deprivation group (%)			
I Most deprived	1,591 (12.96)	2,090 (15.15)	**<0.001**
II	2,063 (16.81)	2,417 (17.52)	
III	2,761 (22.50)	3,196 (23.17)	
IV	4,364 (35.56)	4,542 (32.92)	
V Least deprived	1,274 (10.38)	1,453 (10.53)	
No deprivation data	219 (1.78)	98 (0.71)	

ASA = American Society of Anesthesiologist Score; CCI = Charlson Comorbidity Index; SD = Standard Deviation

*t-test.

**Mann–Whitney *U* test.

Seven centres performed more than ten day cases (Table 3). For these centres, a comparative analysis of cases performed as a day case vs those done as inpatients found that overall there was no difference in patient demographics between the two groups. One centre demonstrated a difference in patient characteristics based on deprivation group.

**Table 3:** Patient demographics (centre level data)

Demographic	Centre 1		*p-*value	Centre 2		*p-*value	Centre 3		*p-*value	Centre 4		*p-*value	Centre 5		*p-*value	Centre 6		*p-*value	Centre 7		*p-*value
	D, *n* = 18	N, *n* = 107		D, *n* = 16	N, *n* = 734		D, *n* = 15	N, *n* = 157		D, *n* = 13	N, *n* = 199		D=14	N=87		D=25	N=32		D=13	N=112	
Age (sd)	73.79 (7.30)	74.61 (6.96)	0.65	70.63 (8.75)	72.94 (8.41)	0.28	73.51 (9.73)	74.87 (7.43)	0.51	72.59 (4.07)	75.90 (7.80)	0.13	75.14 (5.52)	74.68 (7.86)	0.83	74.69 (6.80)	74.93 (5.63)	0.97	73.92 (8.50)	74.82 (73.50)	0.67
Gender																					
Male	8	26	0.08	8	254	0.20	4	45	0.87	4	62	0.98	6	26	0.33	9	11	0.90	5	45	0.91
Female	10	81		8	480		11	112		9	137		8	61		16	21		8	67	
ASA																					
1	0	1	0.148	1	44	0.38	0	4	0.62	1	9	0.36	1	0	0.08	5	1	0.11	0	0	0.33
2	6	61		13	448		8	65		10	110		9	52		15	25		12	109	
3	12	45		2	241		6	83		2	78		4	34		5	6		1	3	
4 + 5	0	0		0	1		1	5		0	2		0	1		0	0		0	0	
Mean CCI (sd)	4.56 (1.72)	4.31 (2.04)	0.32	4.25 (1.95)	4.12 (2.01)	0.99	4.67 (1.72)	4.88 (2.36)	0.74	3.92 (1.12)	5.16 (2.32)	0.08	3.86 (1.92)	4.67 (2.30)	0.11	4.08 (2.00)	4.00 (1.90)	0.96	3.85 (1.14)	4.13 (1.84)	0.78
Deprivation group																					
I	0	3	0.55	1	106	**0** **.** **02**	5	41	0.21	2	27	0.89	11	47	0.29	3	5	0.15	1	15	0.75
II	1	10		5	132		3	36		2	38		3	17		6	5		4	22	
III	7	23		5	142		1	34		4	58		0	10		8	4		4	28	
IV	7	46		2	274		3	37		5	63		0	11		8	14		4	41	
V	3	25		3	35		3	9		0	13		0	2		0	4		0	6	
No deprivation data	0	0		0	45		0	0		0	0		0	0		0	0		0	0	

ASA = American Society of Anesthesiologist Score; CCI = Charlson Comorbidity Index; D = day case; N = non-day case

**Figure 2:**
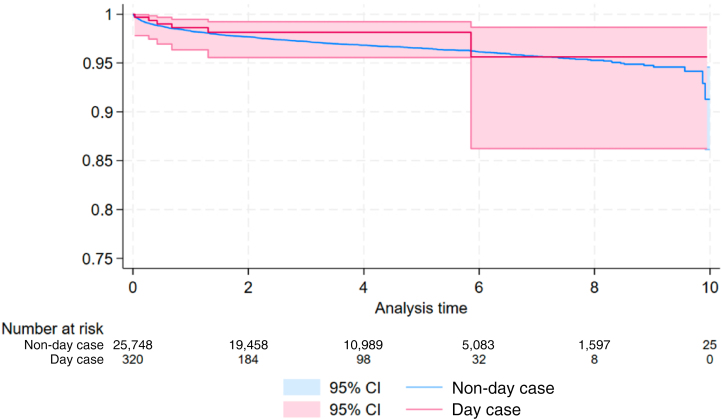
Kaplan–Meier curve for implant survival

The Kaplan–Meier curve for implant survival in the population of day cases vs non-day cases is presented in Figure 2.

The cumulative risk of revision for both day case and non-day case patients is presented in Table 4.

**Table 4:** Cumulative revision rates

	Cumulative revision by year % (95% confidence interval)				
	1 year	3 year	5 year	7 Year	10 year
Day case	1.45 (0.55–3.83)	1.93 (0.80–4.61)	1.93 (0.80–4.61)	3.96 (1.34–11.40)	3.96 (1.34–11.40)
Non-day case	1.76 (1.60–1.94)	2.84 (2.62–3.06)	3.53 (3.27–2.80)	4.35 (4.01–4.73)	5.28 (4.71–5.91)

In the day case group, one patient had a non-revision reoperation (0.31%), which was a MUA ± capsulotomy. In the non-day case group, 217 (0.84%) patients underwent a total of 228 non-revision reoperations; these included 11 stabilisations (4.82%), 39 relocations (17.11%), 138 MUA ± capsulotomies (60.53%), 11 synovectomies (4.82%) and 29 fracture fixations (12.72%).

In the day case group, five patients had a total of six medical complications within 30 days that required readmission (1.56%); these included four episodes of lower respiratory tract infection, one myocardial infarction (MI) and one urinary tract infection (UTI). Of the non-day case patients, 1,633 (6.34%) had a total of 1,936 postoperative complications. This included 82 pulmonary emboli (PE) (4.25%), 32 deep vein thromboses (DVT) (1.65%), 28 myocardial infarctions (1.45), 669 lower respiratory tract infections (34.56), 705 acute kidney injuries (36.42), 395 urinary tract infections (20.40%), and 25 cerebrovascular events (1.29%).

The validated PROMs database was linked to the NJR database and 144 patients had at least one OSS score reported in the day case group (45.00%) and 12,726 (49.43%) in the non-day case group. Thirty-four (10.62%) patients had paired preoperative to 6-month outcomes in the day case group and 2,335 (9.07%) in the non-day case group. There was a mean increase of 19.83 (11.32) in the day case group and a mean increase of 19.16 in the non-day case group (11.80). In the day case group, 88.24% of patients had an increase in OSS that reached MCID and 88.58% achieved this in the non-day case group. The mean OSS in each group at specific follow-up intervals is presented in Table 5.

**Table 5:** Mean Oxford Shoulder Score

	Preoperative	6 month	3 year	5 year
Day case	16.96 (8.35)	36.32 (11.52)	38.00 (8.28)	40.11 (10.95)
Non-day case	15.38 (8.52)	34.14 (10.98)	35.57 (11.07)	35.53 (11.30)

## Discussion

The use of RSA has increased substantially in the UK and this pattern has been mirrored worldwide.^2,16,17^ The trend is predicted to continue, with a projection that the use of shoulder arthroplasty will increase by 234% by 2050 in England, with a predicted annual cost of £235 million.^18^ A reduction in length of stay for hip and knee arthroplasty has been shown to have significant financial benefits with no detriment to patient safety, and in the United States, a study on ambulatory total shoulder arthroplasty (TSA) found low complication and readmission rates as well as significant cost savings.^19–21^ Key aspects in reducing length of stay include patient selection and education, as well as evidence- and consensus-driven clinical pathways.^22^ In England, there is established guidance on achieving day case surgery for hip and knee arthroplasty and this contains evidence-based information on patient selection, preoperative and postoperative care, as well as infrastructure guidance to enable the facilitation of day case surgery.^23^ At present no such guidance exists for shoulder arthroplasty. With expansion in use of shoulder arthroplasty and increasing pressure on healthcare services, day case shoulder arthroplasty could provide a reduction in the inevitable burden on services.

Data from the NJR in this study show an increase in day case procedures year on year except for 2020, which reflects a lower overall number of arthroplasty cases because of the COVID-19 pandemic. Day case RSA cases made up 1.23% of the total cohort. Day case patients were more likely to be younger and male compared with the non-day case cohort. Patients also had a lower CCI and ASA score in the day case group. When reviewing demographics in centres that provided day case RSA against those that did not, these centres had a higher proportion of male patients, had fewer comorbid patients and the centres were in less-deprived areas. Looking at specific centres that provided more than ten day cases, there was no significant difference in patient demographics between day case and non-day case patients; however, case numbers were small and therefore this limits the ability to draw conclusions from this analysis. In order to develop day case practice it may be pertinent to reduce variation in patient selection, developing selection criteria for patients who may benefit from day case arthroplasty. From the demographic analysis this may involve, younger more medically fit patients.

Day case patients had a revision rate of 1.45% at 1 year, 1.93% at 3 and 5 years, and 3.96% at 7 years. Non-day case patients had a revision rate of 1.76% at 1 year, 2.84% at 3 years, 3.53% at 5 years and 4.35% at 7 years. In total, 1.56% of the day case patients required readmission for a medical complication within 30 days compared with 6.34% of non-day case patients. There was a mean increase in OSS preoperatively to 6 months of 19.83 (11.32) in the day case group and 19.16 in the non-day case group (11.80). In the day case group, 88.24% of patients had an increase in OSS that reached MCID and 88.58% achieved this in the non-day case group; however, the number completing OSS scoring at 6 months was low.

Current literature on day case RSA is limited. The study by Tansey *et al* reviewed 21 patients undergoing day case RSA, had a similar average age of 74 years and found an increase in OSS of 16 to 31 at 6 months, which was slightly lower than the current study (36.32) but demonstrated a high patient satisfaction rate, with 81% of patients saying they would undergo the procedure as a day case again.^3^ Fournier *et al* used an algorithm to select patients they deemed suitable for outpatient shoulder arthroplasty (TSA and RSA). They included age <70 years, no pulmonary or cardiac comorbidity and no history of DVT/PE as selection criteria. In their cohort of 61 patients, 1 (2%) required a secondary operation (haematoma evacuation). Implementation of day case shoulder arthroplasty in the UK has been shown to be successful in one stand-alone day case unit with a designated pathway based on preoperative assessment.^24^ This study initially implemented the pathway for hemiarthroplasty patients and because of successful implementation expanded to TSA and showed that day case surgery was safe and comparable with inpatient arthroplasty.^24^ Other studies in the literature have mainly focused on risk predictors for prolonged length of stay and readmission following day case TSA.^21,25^ These studies highlight risk factors for readmissions such as obesity, diabetes mellitus, vascular disease, and cardiac, renal and lung disease, and emphasise the need for appropriate patient selection when considering day case arthroplasty. This analysis of outcomes from the NJR adds to what was a small body of evidence suggesting that outcomes for day case RSA are similar if not better than the general RSA population with a high patient satisfaction rate.

### Study limitations

A limitation of this study is that merging the databases, because of a lack of HES data for all patients as well as being limited to England, reduced the sample size, which remains small in comparison with non-day case procedures, and this limits the ability to compare statistically with sufficient power. There is also no information on the timing of surgery, postoperative protocols or medical assessments for the day case patients. Although we were able to capture readmissions for medical complications to hospital, this study is unable to review whether a patient attended a general practitioner or required extra or earlier follow-up. This study, however, remains the largest for day case RSA in the literature.

## Conclusion

This study has found that using national registry data, day case RSA has low revision, reoperation and complication rates as well as patient satisfaction comparable with that of non-day case patients. This underlines the safety of current practices for day case RSA with appropriate case selection, and the importance of developing this practice from a health policy perspective.
